# Protein-Protein Interactions Prediction Using a Novel Local Conjoint Triad Descriptor of Amino Acid Sequences

**DOI:** 10.3390/ijms18112373

**Published:** 2017-11-08

**Authors:** Jun Wang, Long Zhang, Lianyin Jia, Yazhou Ren, Guoxian Yu

**Affiliations:** 1 College of Computer and Information Science, Southwest University, Chongqing 400715, China; kingjun@swu.edu.cn (J.W.); 18234031968@163.com (L.Z.); 2 College of Information Engineering and Automation, Kunming University of Science and Technology, Kunming 650000, China; jlianyin@163.com; 3 SMILE (Statistical Machine Intelligence & Learning) Lab and Big Data Research Center, School of Computer Science and Engineering, University of Electronic Science and Technology of China, Chengdu 610000, China; yazhou.ren@uestc.edu.cn

**Keywords:** protein-protein interactions, amino acid sequences, local conjoint triad descriptor, deep neural networks

## Abstract

Protein-protein interactions (PPIs) play crucial roles in almost all cellular processes. Although a large amount of PPIs have been verified by high-throughput techniques in the past decades, currently known PPIs pairs are still far from complete. Furthermore, the wet-lab experiments based techniques for detecting PPIs are time-consuming and expensive. Hence, it is urgent and essential to develop automatic computational methods to efficiently and accurately predict PPIs. In this paper, a sequence-based approach called DNN-LCTD is developed by combining deep neural networks (DNNs) and a novel local conjoint triad description (LCTD) feature representation. LCTD incorporates the advantage of local description and conjoint triad, thus, it is capable to account for the interactions between residues in both continuous and discontinuous regions of amino acid sequences. DNNs can not only learn suitable features from the data by themselves, but also learn and discover hierarchical representations of data. When performing on the PPIs data of *Saccharomyces cerevisiae*, DNN-LCTD achieves superior performance with accuracy as 93.12%, precision as 93.75%, sensitivity as 93.83%, area under the receiver operating characteristic curve (AUC) as 97.92%, and it only needs 718 s. These results indicate DNN-LCTD is very promising for predicting PPIs. DNN-LCTD can be a useful supplementary tool for future proteomics study.

## 1. Introduction

Protein-protein interactions (PPIs) play critical roles in virtually all cellular processes, including immune response [1], DNA transcription and replication [2], and signal transduction [3]. Therefore, correctly identifying PPIs can not only better elucidate protein functions but also further understand the various biological processes in cells [4,5,6]. In recent years, biologists take advantage of high-throughput technologies to detect PPIs, such as mass spectrometric (MS), tandem affinity purification (TAP) [7], yeast two-hybrid system (Y2H) [8,9], and so on. Unfortunately, these wet-lab experiments are costly and labor-intensive, and have a high rate of both false positive and false negative, and limited coverage. Hence, it is extremely imperative to develop reliable computational models to predict PPIs in large scale [10].

So far, a number of computational methods have been developed for the detection of PPIs. Most of these methods are based on the genomic information, such as Gene Ontology and annotations [11], phylogenetic profile, and gene fusion [12]. Methods employ 3D structural information of proteins [13,14] and the sequence conservation between interacting proteins [15] also have been reported. However, these methods are heavily dependent on the pre-knowledge of the proteins, such as protein functional domains, structure information of proteins, and physicochemical properties of proteins [16,17]. In other words, all these methods are hardly implementable unless the pre-knowledge about proteins is available. Compared to the abundant data of protein sequences, other types of data including 3D structure, Gene Ontology annotations, and domain-domain interactions of proteins are still limited.

Many researchers have innovated sequence-based methods for detecting PPIs [18,19,20,21,22,23,24], and experimental results have shown that the information of the amino acid sequences alone is sufficient to identify new PPIs. Among them, Shen et al. [18] achieved an excellent effect based on support vector machine (SVM). They grouped 20 standard amino acids into 7 classes according to their dipoles, volumes of the side chains, and then employed conjoint triad (CT) method to extract the features information of amino acid sequences based on the classification of amino acids. Next, SVM predictor is used to predict PPIs. Their method yields a high prediction accuracy of 89.3% on human PPIs. However, it does not consider the neighboring effect and PPIs are almost always occurring in the non-continuous segments of amino acid sequences. Guo et al. [19] developed SVM-based method by using auto covariance (AC) to abstract the feature information in the discontinuous amino acid segments in the sequence, and obtained a perfect result with accuracy as 86.55% on *Saccharomyces cerevisiae (S. cerevisiae)*. Yang et al. [20] introduced local descriptor (LD) to encode amino acid sequences based on *k*-nearest neighbor (*k*NN). In this study, they grouped 20 standard amino acids into 7 classes as done by Shen et al. [18]. Then they divided an entire protein sequence into ten segments with varying length and extracted information of each segment. Finally, they applied *k*NN to predict PPIs. This *k*NN based method achieves prediction accuracy as 86.15% on *S. cerevisiae*. You et al. [21] innovated a novel multi-scale continuous and discontinuous (MCD) descriptor based on the LD [20]. In order to discover more information from amino acid sequences, MCD descriptor applies the binary coding scheme to construct varying length segments and abstracts the feature vectors from these segments. Then the minimum redundancy maximum relevancy criterion [25], which can reduce the feature abundance and computation complexity, is used to select an optimal feature subset. Finally, SVM is employed to predict new PPIs. This solution obtains a high accuracy as 91.36% on *S. cerevisiae*. Recently, Du et al. [22] employed deep neural networks (DNNs), a recently famous and popular machine learning technique, and amphiphilic pseudo amino acid composition (APAAC) [26] to predict new PPIs. They firstly extracted the feature information from two respective amino acid sequences by APAAC, then they took APAAC features of two respective proteins as inputs of two separate DNNs and fused the two DNNs to predict PPIs. Their method obtains an accuracy of 92.5% on PPIs of *S. cerevisiae*.

LD descriptor [20] only considers the neighboring effect of adjacent two types of amino acids. Hence, it cannot sufficiently abstract information of neighboring amino acids but can sufficiently discover information of discontinuous segments of the amino acid sequences. On the other hand, CT [18] considers the neighboring effect of adjacent three types of amino acids but ignores the discontinuous information. Given these observations, we combine the advantage of local descriptor [20] and conjoint triad method [18], and introduce a novel feature representation method called local conjoint triad descriptor (LCTD). LCTD can better account for the interactions between sequentially distant but spatially close amino acid residues than LD [20] and CT [18]. DNNs, a recently powerful machine learning technique, can not only reduce the impact of noise in the raw data and automatically extract high-level abstractions, but also have better performance than traditional models [27,28]. Inspired by these characteristics of DNNs, we employ DNNs to detect the PPIs based LCTD feature representation of amino acid sequences and introduce an approach called DNN-LCTD. Particularly, DNN-LCTD extracts the feature information of the amino acid sequences by LCTD, then it trains a 3-hidden layers neural network by taking feature sets derived from LCTD as inputs and accelerates training by graphics processing unit (GPU). Finally, the learned network is employed to predict new PPIs. We perform experiments on PPIs of *S. cerevisiae*, DNN-LCTD achieves 93.12% accuracy, 93.83% sensitivity, 93.75% precision, and area under the receiver operating characteristic curve (AUC) as 97.92%, and only uses 718 s. Experimental results on other five independent datasets: *Caenorhabditis elegans* (4013 interacting pairs), *Escherichia coli* (6954 interacting pairs), *Helicobacter pylori* (1420 interacting pairs), *Homo sapiens* (1412 interacting pairs), and *Mus musculus* (313 interacting pairs), further demonstrate the effectiveness of DNN-LCTD.

## 2. Results and Discussion

In this section, we briefly introduce the evaluation metrics employed in performance comparison. Then, we provide the recommended configuration of experiments. Finally, we analyze and discuss the experimental results and compare our results with those of other related work.

### 2.1. Evaluation Metrics

To reasonably evaluate the performance of DNN-LCTD, five-fold cross validation is adopted. Cross validation can avoid the overfitting and enhance the generalization performance [29]. Six evaluation metrics are used to quantitatively measure the prediction performance of DNN-LCTD, including overall prediction accuracy (ACC), precision (PE), recall (RE), specificity (SPE), matthews correlation coefficient (MCC), $F_{1}$ score values, and area under the receiver operating characteristic curve (AUC). They (except AUC) are defined as follows:

(1) $$\mathrm{ACC}=\frac{\mathrm{TP}+\mathrm{TN}}{\mathrm{TP}+\mathrm{FP}+\mathrm{TN}+\mathrm{FN}}$$

(2) $$\mathrm{PE}=\frac{\mathrm{TP}}{\mathrm{TP}+\mathrm{FP}}$$

(3) $$\mathrm{RE}=\frac{\mathrm{TP}}{\mathrm{TP}+\mathrm{FN}}$$

(4) $$\mathrm{SPE}=\frac{\mathrm{TN}}{\mathrm{TN}+\mathrm{FP}}$$

(5) $$\mathrm{MCC}=\frac{\mathrm{TP}\times \mathrm{TN}-\mathrm{FP}\times \mathrm{FN}}{\sqrt{\left(\mathrm{TP}+\mathrm{FP})(\mathrm{TP}+\mathrm{FN})(\mathrm{TN}+\mathrm{FP})(\mathrm{TN}+\mathrm{FN}\right)}}$$

(6) $$F_{1}=\frac{2\mathrm{TP}}{2\mathrm{TP}+\mathrm{FP}+\mathrm{FN}}$$

where TP (true positive) is the number of the true PPIs that are correctly predicted, the FN (false negative) is the number of the true interacting pairs that are failed to be predicted, TN (true negative) is the number of the true non-interactions protein pairs of that are correctly predicted, FP (false positive) is the number of true non-interactions pairs that are failed to be predicted. MCC is a measure for the quality of binary classification. MCC equal to 0 means completely random prediction, −1 means completely wrong prediction and 1 means perfect prediction. $F_{1}$ score is a harmonic average of precision and recall. A larger $F_{1}$ denotes a better performance. Receiver operating characteristic curve (ROC) can elucidate the diagnostic ability of a binary classifier system by graphical plot. This curve is produced by plotting the true positive rate versus the false positive rate under different thresholds [30,31]. AUC is the area under the ROC curve and its value is widely employed to compare predictors. The larger the value of AUC, the better the predictor is.

### 2.2. Experimental Setup

**Figure 1:**
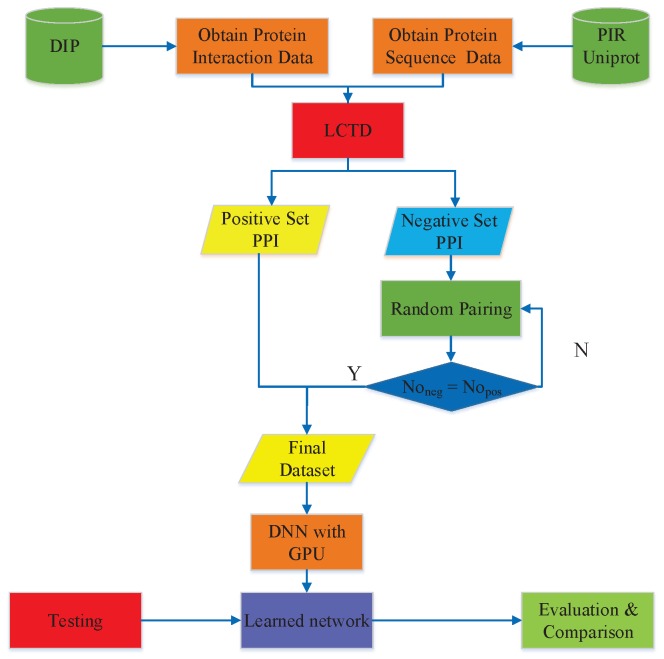
The flowchart of DNN-LCTD for predicting protein-protein interactions. There are some abbreviations in this figure, including database of interacting proteins (DIP), protein information resource, local conjoint triad descriptor (LCTD), protein-protein interactions (PPIs), and graphics processing unit (GPU). The ${\mathrm{No}}_{\mathrm{neg}}$ is the number of non-interacting protein pairs, ${\mathrm{No}}_{\mathrm{pos}}$ is the number of interacting protein pairs. Y/N means yes/no.

DNN-LCTD is implemented on Tensorlfow platform https://www.tensorflow.org. The flowchart of DNN-LCTD is shown in Figure 1. DNN-LCTD firstly encodes the amino acid sequences using the novel LCTD. After that, we train a 3-hidden layers neural network with GPU based on the encoded feature sets. Finally, we apply the learned DNN to predict new PPIs. Hyper-parameters of the DNN model heavily impact the experimental results. Deep learning algorithms have ten or more hyper-parameters to be properly specified, trying all of them is impossible in practice [32]. We summarize the recommended configuration of DNN-LCTD in Table 1. As to the parameters setup of the comparing methods, we use the grid search approach to obtain the optimal parameters. The optimal parameters is shown in Table 2. The details of the parameters of comparing methods are available at http://scikit-learn.org. For Du et al. work [22], there are too many parameters need to be set, the information of parameters can be accessed via http://ailab.ahu.edu.cn:8087/DeepPPI/index.html. All the experiments are carried out on a server with configuration: CentOS 7.3, 256 GB RAM, and Intel Exon E5-2678 v3. DNN-LCTD uses NVIDIA Corporation GK110BGL [Tesla K40c] to accelerate training of DNNs.

### 2.3. Results on PPIs of S. cerevisiae

In order to achieve good experimental results, the corresponding hyper-parameters for deep neural network are firstly optimized. Table 1 provides the recommended hyper-parameters that are chosen by a large number of experiments. Considering the numerous samples used in this work, five-fold cross validation is adopted to reduce the impact of data dependency and to minimize the risk of over-fitting. Thus, five models are generated for the five sets of data. Table 3 reports the results of DNN-LCTD on five individual folds (fold 1–5) and the overall average results of five folds. From Table 3, we can observe that all the prediction accuracies are nearly ≥93.1%, the precisions are ≥93.35%, all the recalls are almost ≥93.4%, the specificities are ≥92.75%, and the $F_{1}$ are ≥92.4%. In order to comprehensively evaluate the performance of DNN-LCTD, the MCC and AUC are also calculated. DNN-LCTD achieves superior prediction performance with an average accuracy as 93.11%, precision as 93.75%, recall as 92.40%, specificity as 92.75%, MCC as 86.24%, $F_{1}$ as 93.06%, and AUC as 97.95%.

Plenty sequence-based methods have been employed to predict PPIs. We compare the prediction performance of DNN-LCTD with the other existing approaches on *S. cerevisiae*, including Guo et al. [19], Yang et al. [20], Zhou et al. [33], You et al. [21], and Du et al. [22]. The details of these approaches were introduced in Section 1. From Table 3, we can observe that DeepPPI [22] achieves the best performance among comparing methods (except DNN-LCTD). DeepPPI firstly uses APAAC descriptor to encode the amino acid sequence for each protein and takes the APAAC features as separate inputs for two individual DNNs to extract high-level features of these two proteins, it finally fuses the extracted features to predict PPIs. Its average prediction accuracy is 92.58% ± 0.38%, precision is 94.21% ± 0.45%, recall is 90.95% ± 0.41%, MCC is 85.41% ± 0.76%, $F_{1}$ is 92.55% ± 0.39%, and AUC is 97.55% ± 0.16%. This result mean that DeepPPI [22] is indeed successful for predicting new PPIs using DNNs with APAAC [26]. DNN-LCTD encodes the amino acid sequences of each protein via LCTD descriptor, it then concatenates the LCTD features of two proteins into a longer feature vector and takes the concatenated features as inputs of DNN for prediction. The average accuracy, recall, MCC, $F_{1}$ and AUC of DNN-LCTD are 0.53%, 1.45%, 0.83%, 1.05% and 0.4% higher than those of DeepPPI, respectively. The reason is that LCTD can discover more feature information from amino acid sequences than APAAC. The DNN-LCTD is far greater than other comparing approaches can be attributed to the merits of DNNs and of LCTD. The contributions of LCTD and DNNs will be further investigated in Section 2.4 and Section 2.5. The *S. cerevisiae* dataset contains tremendous samples, hence, a little improvement in prediction performance still has a great effect. Based on these experimental results, we can conclude that DNN-LCTD can more effectively predict PPIs than other comparing methods, and the proposed LCTD descriptor can explore more patterns from continuous and discontinuous amino acid segments.

The adopted negative PPIs set may lead to a biased estimation of prediction performance [34]. To prove the rationality of a negative set generated by selecting non-interacting pairs of non-co-localized proteins [19], we perform additional testing on a simulated dataset of *S. cerevisiae*. Particularly, we firstly construct the negative PPIs set by pairing proteins whose subcellular localizations are different, and we randomly select 17,257 protein pairs as the negative set of the simulated dataset. Next, we construct the positive PPIs set by pairing proteins whose subcellular localizations are the same, regardless of being interacting pairs or not. We then randomly select 17,257 protein pairs as the positive set. As a result, the simulated testing dataset includes 34,514 protein pairs for testing, where half are positives and the other half are negatives. After that, we randomly divide these testing PPIs into five folds, and apply the same DNN as trained on the dataset in Table 3 to predict PPIs in each fold. Table 4 reports the evaluation results on this simulated dataset. From Table 4, we can see that the values of accuracy, recall, MCC, and $F_{1}$ are much lower than the corresponding values reported in Table 3. The reason for the high specificity in Table 4 is that the way of constructing negative dataset in the training dataset (used in Table 3) and simulated testing dataset is the same. These results indicate that the constructed negative set is reasonable.

### 2.4. Comparison with Different Descriptors

**Figure 2:**
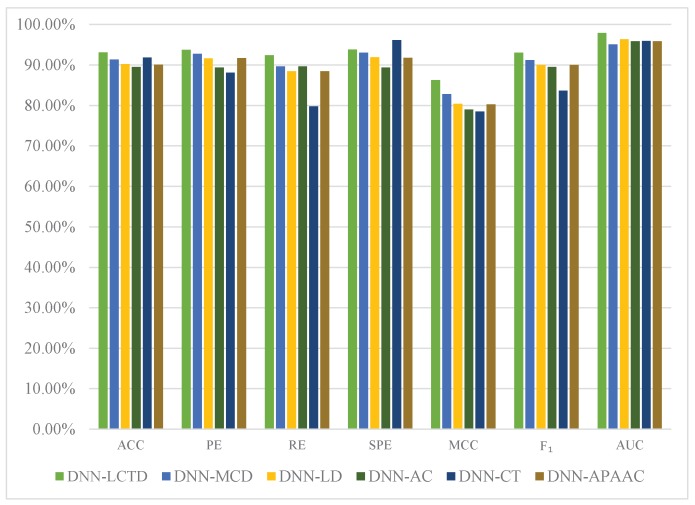
Performance comparison based on DNNs with AC, LD, MCD, LCTD, CT, or APAAC on *S. cerevisiae* dataset.

To further investigate the contribution of the novel local conjoint triad descriptor, we separately train DNNs based on CT [18], AC [19], LD [20,33], MCD [21], APAAC [22], and LCTD. After that we use pairwise *t*-test at 95% significance level to check the statistical significance between LCTD and LD, MCD, AC, CT, APAAC in five-fold cross validation and report the results in Figure 2 and Table 5. In Table 5, • means that LCTD is statistically significant better than other descriptors on a particular evaluation metric. From Figure 2 and Table 5, we can observe that the prediction performance using LCTD outperforms other descriptors across nearly all evaluation metrics. The ACC, MCC, $F_{1}$ and AUC of DNN-LCTD are 1.76%, 3.48%, 1.86%, and 2.85% higher than those of DNN-MCD; 2.92%, 5.81%, 3.05% and 1.62% higher than those of DNN-LD; 3.62%, 7.25%, 3.56% and 2.06% than those of DNN-AC; 1.27%, 7.74%, 9.41% and 1.99% than those of DNN-CT; 3.02%, 5.99%, 3.03% and 2.06% than those of DNN-APAAC, respectively. These improvements can be attributed to that LCTD can extract more useful feature information of amino acid sequences by incorporating the advantage of LD [20,33] and conjoint triad (CT) descriptor [18]. From these results, we can conclude that the novel LCTD can more sufficiently capture the feature information of amino acid sequences for PPIs prediction.

### 2.5. Comparison with Existing Methods

**Figure 3:**
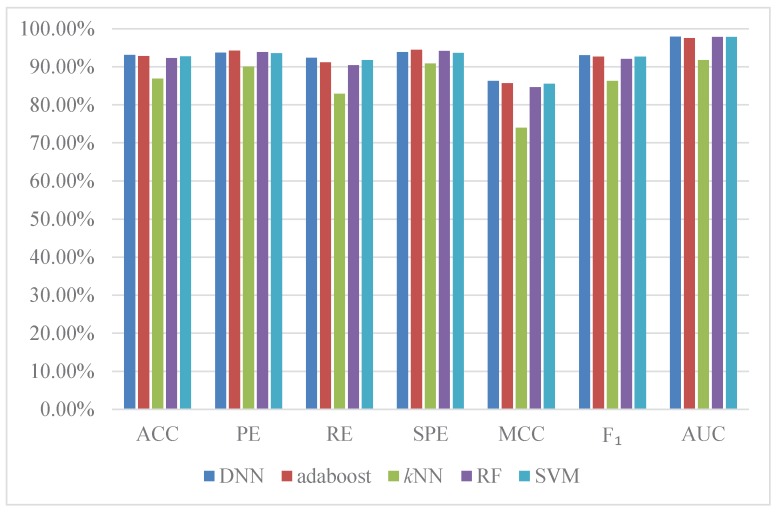
Performance comparison of other algorithms with LCTD descriptor on *S. cerevisiae* dataset.

Meanwhile, in order to further investigate the effective of DNNs, we separately train the different state-of-the-art predictors on *S. cerevisiae* dataset using LCTD to encode amino acid sequences, these predictors include support vector machine (SVM) [35], *k* neighbor nearest (*k*NN) [36], random forest (RF) [37], and adaboost [38]. Then, we compare the prediction performance based on the six already introduced evaluation metrics. In this study, five-fold cross validation is employed to reduce the impact of data dependency and enhance the reliability of the experiments. The results are shown in Figure 3. From Figure 3 we can see that a high average accuracy of 93.11% is obtained by DNN-LCTD. The average accuracy of adaboost, *k*NN, random forest, and SVM are 92.83%, 86.87%,92.28%, 92.76%, respectively. DNNs have the highest prediction performance across all evaluation metrics except in RE and SPE. In practice, grid search is used to seek the optimal parameters of these comparing algorithms. We also show the training speed of different comparing methods in Table 6. We can observe that DNN-LCTD with central processing unit (CPU) is separately 2, 25 and 39 times faster than random forest, adaboost and SVM. In order to speed up training of DNN-LCTD, GPU is employed. We can see that the training time of DNN-LCTD with GPU is 3 times faster than that with CPU, 4, 9.5, 97.5 and 148 times than *k* neighbor nearest, random forest, adaboost and SVM. According to these experimental results, we can conclude that DNN-LCTD can accurately and efficiently predict PPIs from amino acid sequences.

### 2.6. Results on Independent Datasets

To further assess the practical prediction ability of DNN-LCTD and other comparing methods, we firstly train different models with optimal configurations (details in Section 2.2) using PPIs of *S. cerevisiae* dataset (34,514 protein pairs). After that, five independent datasets that only contain the samples of interactions, including *Caenorhabditis elegans* (4013 interacting pairs), *Escherichia coli* (6954 interacting pairs), *Helicobacter pylori* (1420 interact-ing pairs), *Homo sapiens* (1412 interacting pairs), and *Mus musculus* (313 interacting pairs), are used as test sets to evaluate the prediction performance of these trained models. The prediction results are shown in Table 7. From Table 7, we can observe that the accuracy of DNN-LCTD on *C. elegans*, *E. coli*, *H. pylori*, *H. sapiens*, and *M. musculus* are 93.17%, 94.62%, 87.38%, 94.18%, and 92.65%, respectively. DNN-LCTD has a higher accuracy than DeepPPI [22] and SVM + LD [33] on *E. coil*, *H. sapiens*, and *M. musculus*. The accuracy of SVM + LD [33] is far lower than DNN-LCTD on *C. elegans* and *H. pylori*. These prediction accuracies are satisfying except on *H. pylori*. The reason is that we use *S. cerevisiae* as the training set to train models, the trained model is inclined to species that are closer to *S. cerevisiae*. In reality, *S. cerevisiae* has closer relationship with other four datasets than with *H. pylori*. These prediction results indicate that DNN-LCTD has a good generalization ability for predicting PPIs.

## 3. Materials and Methods

In this section, we briefly introduce the datasets we used for experiments, including *S. cerevisiae* and other five independent datasets. Then, we introduce the details of LCTD, a novel feature representation descriptor. Finally, we present a brief introduction of deep neural networks (DNNs), including characteristics and skills.

### 3.1. PPIs Datasets

To reliably evaluate the performance of DNN-LCTD, a validation benchmark dataset is necessary. We adopt the *S. cerevisiae* dataset used by Du et al. [22] for experiments. This dataset was collected from the database of interacting proteins (DIP; version 20160731) [39]. The protein pairs of this dataset exclude proteins with fewer than 50 amino acids and ≥40% sequence identity [19]. Finally, this dataset contains 17,257 positive protein pairs. Negative examples impact the prediction results of PPIs. The common approach is based on annotations of cellular localization [40,41]. The negative set is obtained by pairing proteins whose subcellular localizations are different. The strategy must meet the following requirements [18,19]: (1) the non-interaction pairs cannot appear in the positive dataset, and (2) the contribution of proteins in the negative set should be as harmonious as possible, which means that proteins without subcellular localization information, or denoted as ’putative’, ’hypothetical’ are excluded for constructing the negative set. Finally, 48,594 negative pairs are generated via this strategy. In the end, *S. cerevisiae* contains 34,514 protein pairs, where half are from positive dataset and the other (17,257 negative pairs) are randomly selected from the whole negative set. Other five independent PPIs datasets, including *Caenorhabditis elegans* (4013 interacting pairs), *Escherichia coli* (6954 interacting pairs), *Helicobacter pylori* (1420 interacting pairs), *Homo sapiens* (1412 interacting pairs), and *Mus musculus* (313 interacting pairs) [33], are used as independent test datasets to assess the generalization ability of DNN-LCTD. These datasets are available at http://ailab.ahu.edu.cn:8087/DeepPPI/index.html.

### 3.2. Feature Vector Extraction

Whether the encoded features are reliable or not can heavily affect the performance of PPIs prediction. The main challenge is how to effectively describe and represent an interacting protein pairs by a fixed length feature vector, in which the essential information content of interacting proteins is fully encoded. Various sequence-based methods are proposed to predict new PPIs, but one flaw of them is that they cannot adequately capture interaction information from continuous and discontinuous amino acid segments at the same time. To overcome this problem, we introduce a novel local conjoint triad descriptor (LCTD), which incorporates the advantage of local descriptor (LD) [20,33] and conjoint triad (CT) [18] sequence representation approach. To clearly introduce the LCTD, we first briefly introduce the feature representation methods of CT [18] and LD [20,33] in the following two subsections.

#### 3.2.1. Conjoint Triad (CT) Method

Shen et al. [18] introduced the conjoint triad (CT). In order to conveniently represent the 20 standard amino acids and to suit synonymous mutation, they firstly divided these 20 standard amino acids into 7 groups based on the dipoles and volumes of the side chains as shown in Table 8. After that, the conjoint triad method is introduced to extract the sequence information, which includes the properties of one amino acid and its vicinal amino acids and regards any three continuous amino acids as a unit [18]. The process of generating descriptor vectors is described as follows.

**Figure 4:**
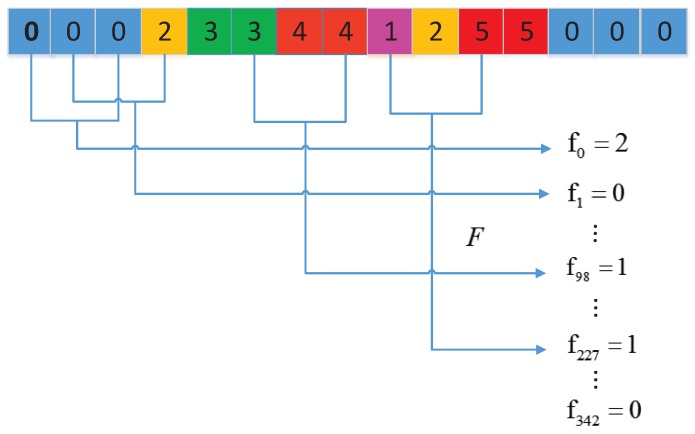
Schematic diagram for conjoint triad. The number is the classes grouped by the dipoles and volumes of the side chains. $f_{i}$ is the frequency that triad type appears in the protein sequence. F is the vector set for all $f_{i}$.

Firstly, they replaced each amino acid in the protein sequence by the index depending on its grouping. For instance, protein sequence “VCCPPVCVVCPPVCVPVPPCCV” is replaced by 0112201001220102022110. Then, binary space (V,F) stands for a protein sequence. Here, V is the vector space of the sequence features, and each feature $v_{i}$ represents a kind of triad type [18]. For example, $v_{1}$, $v_{7}$, and $v_{10}$ are separately representing the triad unit of 100, 010, 310. F is the frequency vector corresponding to V, and the value of the *i*th dimension of F ($f_{i}$) is the frequency of type $v_{i}$ appearing in amino acid sequence [18]. As the amino acids grouped into seven classes, the size V should be 7 × 7 × 7; therefore, i=0,1,⋯,342. The detailed definition and description is shown in Figure 4. Clearly, each protein has a corresponding F vector. Nevertheless, the value of $f_{i}$ relates to the length of amino acid sequence. A longer amino acid sequence generally have a larger value of $f_{i}$, which complicates the comparison between two heterogeneous proteins. As such they employed the normalization to solve this problem as follows:

(7) $$d_{i}=\left(f_{i}-\min\left\{f_{0},f_{1},\cdots ,f_{342}\right\}\right)/\max\left\{f_{0},f_{1},\cdots ,f_{342}\right\}$$

where the value of $d_{i}$ is normalized in the range [0, 1]. $f_{i}$ is the frequency of conjoint triad unit $v_{i}$ appearing in the protein sequence. Finally, they connected the vector spaces of two proteins to present the interaction features. Thus, a 686-dimensional vector (343 for each protein) is generated for each pair of proteins.

#### 3.2.2. Local Descriptor (LD)

**Figure 5:**
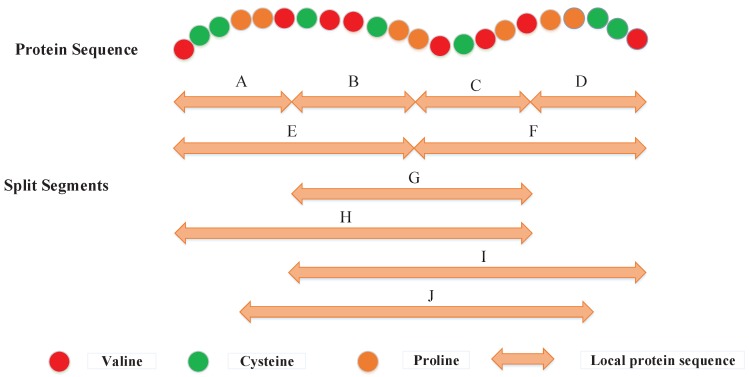
The 10 descriptor regions (A–J) are split for a hypothetical protein sequence. The regions A–D and E–F are obtained by dividing the entire amino acid sequence into four equal regions and two equal regions [20,33], respectively. G stands for the central 50% of the amino acid sequence. Regions H, I, and J represent the first, final and central 75% of the amino acid sequence, respectively.

Local descriptor (LD) is an alignment-free approach previously used to classify several proteins families [42,43]. Yang et al. [20] and Zhou et al. [33] employed this method to extract the interactions information from amino acid sequences. 20 standard amino acids are grouped into 7 groups based on the dipoles and volumes of the side chains at first, as shown in Table 8. Then each entire protein sequence is divided into 10 segments as shown in Figure 5. For each local region, three local descriptors including composition (C), transition (T) and distribution (D) are employed to extract the feature information. C represents the composition of each amino acid group. T stands for the frequency from a type of amino acids to another type. D describes the distribution pattern along the entire region by measuring the location of the first 25%, 50%, 75% and 100% of residues of a given group [33,44].

**Figure 6:**
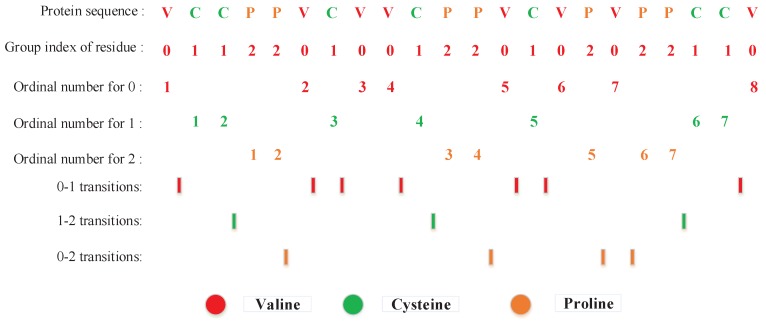
A hypothetical protein sequence figuring the structure of composition, transition and distribution pattern of a protein region.

Then, each local region split is replaced by the index depending on the classification of amino acids. For example, protein sequence “VCCPPVCVVCPPVCVPVPPCCV” is replaced by 0112201001220102022110 based on classification of amino acids as shown in Figure 6. There have eight ‘0’, seven ‘1’, and seven ‘2’ in the protein sequence. The composition for these three symbols is 8 × 100%/(8 + 7 + 7) = 36.36%, 7 × 100%/(8 + 7 + 7) = 31.82%, and 6 × 100%/(8 + 7 + 7) = 31.82%, respectively. There are 7 transitions from ‘0’ to ‘1’ or from ‘1’ to ‘0’ in this sequence, and the percentage frequency of these transitions is (7/21) × 100% = 33.33%. Similarly, the transitions from ‘0’ to ‘2’ or ‘2’ to ‘0’ and transitions from ‘1’ to ‘2’ or ‘2’ to ‘1’ are respectively calculated as (3/21) × 100% = 14.29% and (4/21) × 100% = 19.05%. For distribution D, there are 8 residues encoded as ‘0’ in the example of Figure 6, the position of the first residue ‘0’, the second residue ‘0’ (25% × 8 = 2), the fourth residue ‘0’ (50% × 8 = 4), the sixth ‘0’ residue (75% × 8 = 6), and the eight residue ‘0’ (100% × 8 = 8) in the encoded sequence are 1, 6, 9, 15, and 22, respectively. Thus D descriptor for ‘0’ is: (1/22 × 100% = 4.55%), (2/22 × 100% = 9.09%), (4/22 × 100% = 18.18%), (6/22 × 100% = 27.27%) and (8/22 × 100% = 36.36%), respectively. Similarly, the D descriptor for ‘1’ and ‘2’ is (9.09%, 13.64%, 45.45%, 63.64%, 95.45%) and (18.18%, 22.73%, 54.55%, 72.73%, 86.36%), respectively.

For each local region, three descriptors (C, T, D) are computed and concatenated into a 63-dimensional feature vector, 7 for C, 21 (7 × 6/2) for T and 35 (7 × 5) for D. Then all descriptors from 10 regions are concatenated into an 630-dimensional vector. Finally, LD concatenates the vectors of two individual amino acid sequences. Thus, a 1260-dimensional vector is constructed to characterize each protein pair.

#### 3.2.3. Local Conjoint Triad Descriptor (LCTD)

From the process of LD descriptor [20,33], we can find that it only considers the neighboring effect of adjacent two types of amino acids. Therefore, it cannot sufficiently extract information of neighbor amino acids, but can sufficiently discover information of discontinuous segments of the amino acid sequence. Meanwhile, we observe that the conjoint triad method [18] considers the neighboring effect of adjacent three types of amino acid, but ignores the discontinuous information. Thus, we advocate to integrate the merits of LD [20,33] and conjoint triad (CT) [18] to introduce a novel feature representation of amino acid sequence called LCTD. LCTD groups the 20 standard amino acids into 7 groups on the dipoles and volumes of the side chains at first as shown in Table 8. Then it divides the entire protein sequence into 10 segments as done by LD [20,33]. Next, for each local region, we calculate four descriptors, composition (C), transition (T) and distribution (D), and conjoint triad (CT). C represents the composition of each amino acid group. T stands for the frequency from a type of amino acid to another type. D describes the distribution pattern along the entire region by measuring the location of the first 25%, 50%, 75% and 100% of residues of a given group [33,44]. Conjoint triad considers the properties of one amino acid and its vicinal amino acids, it regards any three continuous amino acids as a unit [18]. These descriptors are introduced in Section 3.2.1 and Section 3.2.2. For each local region, the four descriptors (C, T, D, CT) are calculated and concatenated, and a total of 63 + 343 descriptors are generated: 7 for C, 21 (7 × 6/2) for T and 35 (7 × 5) for D, and 343 for CT. After that, all descriptors from 10 regions are concatenated into an 4060-dimensional vector. Finally, LCTD concatenates the vectors of two individual proteins. Thus, a 8120-dimensional vector is constructed to encode each protein pair. The corresponding equations are shown as follows:

(8) $$D_{Ai}=C⊕T⊕D⊕CT\;\left(i=1,2,\cdots ,10\right)$$

(9) $$D_{Bi}=C⊕T⊕D⊕CT\left(i=1,2,\cdots ,10\right)$$

(10) $$D_{A}=D_{A1}⊕D_{A2}⊕\cdots ⊕D_{A10}$$

(11) $$D_{B}=D_{B1}⊕D_{B2}⊕\cdots ⊕D_{B10}$$

(12) $$D_{AB}=D_{A}⊕D_{B}$$

where *A* and *B* are a pair of proteins, ⊕ is the vector concatenating operator. $D_{A}$, $D_{B}$ is the extracted feature vector from *A* and *B*, respectively. *i* refers to any segment in 10 split segments. $D_{AB}$ is the extracted feature of two amino acid sequences. These 8120-dimensional feature vectors are used as input of DNNs for training and prediction.

### 3.3. Deep Neural Network

Deep learning, a popular type of machine learning algorithms, consists with an artificial neural network of multiple nonlinear layers. It is inspired by the biological neural network that constitutes animal brains. The characteristics of deep learning are that it can learn suitable features from the original data without designed by human engineers, and discover hierarchical representations of data [45]. The depth of a neural network corresponds to the number of hidden layers, and the width is the maximum number of neurons in one of its layers [27]. Neural network with a large number of hidden layers (three or more hidden layers) is called deep neural network [27].

**Figure 7:**
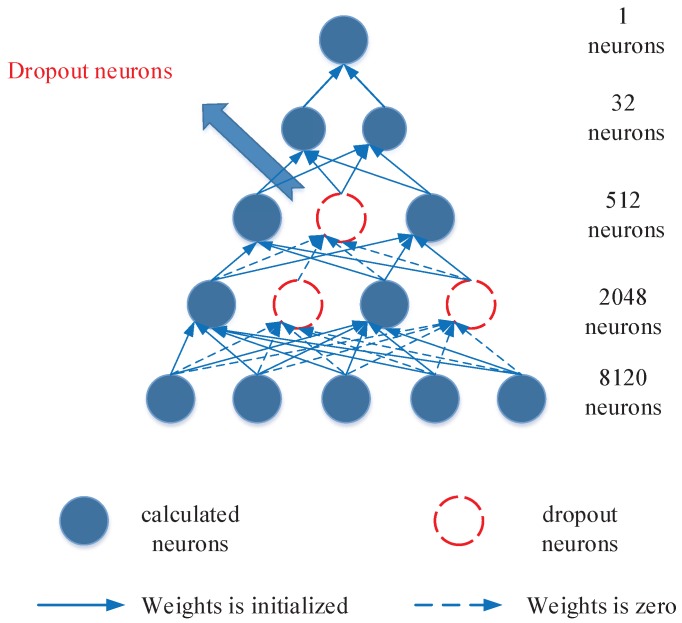
The structure of the adopted DNN with LCTD features and the dropout technique.

The basic structure of DNN consists of an input layer, multiple hidden layers, and an output layer, the special configuration of our neural network is shown in Figure 7. In general, input data (*x*) are given to the DNN, the output values are sequentially computed along the layers of the network. Neurons of a hidden layer or output layer are connected to all neurons of the previous layer [27]. Each neuron computes a weighted sum of its inputs and applies a nonlinear activation function to calculate its outputs f(x) [27]. The representations in the layer below are transformed into slightly more abstract representations by the computation in each layer [46]. In general, the nonlinear activation function including sigmoid, hyperbolic tangent, or rectified linear unit (ReLU) [47]. The sigmoid and ReLU are used in this study.

In this work, we use the mini-batch gradient descent [48] and Adam algorithm [49] to reduce the sensitivity to the specific choice of learning rate [27], and speed up training using GPU. The dropout technique is employed to avoid the overfitting, which the activation of some neurons is randomly set to zero during training in each forward pass as shown in Figure 7 [27]. The dotted line means this neuron will not be activated and calculated. The activation function of ReLU [47] and the loss of cross entropy is employed because they can both accelerate the model training and obtain better prediction results [50]. Batch normalization approach is also employed to reduce the dependency of training with the parameter initialization, speed up training and minimize the risk of over-fitting. The following equations are used to calculate the loss:

(13) $$H_{i1}=\sigma_{1}\left(W_{i1}X_{i1}+b_{i1}\right)\left(i=1,\cdots ,n\right)$$

(14) $$H_{i(j+1)}=\sigma_{1}\left(W_{ij}H_{ij}+b_{ij}\right)\left(i=2,\cdots ,n,\;j=1,\cdots ,h\right)$$

(15) $$L=-\frac{1}{n}\sum_{i=1}^{n}[y_{i}ln\left(\sigma_{2}\left(W_{ih}H_{ih}+b_{ih}\right)+\left(1-y_{i}\right)ln\left(1-\sigma_{2}\left(W_{ih}H_{ih}+b_{ih}\right)\right)\right]$$

where *n* is the number of PPIs for batch training. $\sigma_{1}$ is the activation function of ReLU, $\sigma_{2}$ is the activation function of the output layer with sigmoid, **X** is the batch training inputs, **H** is the outputs of hidden layer, and **y** is the corresponding desired outputs. *h* is the depth of the DNN, **W** is the weight matrix between the input layer and the output layer and **b** is the bias.

## 4. Conclusions

In this article, we propose an efficient approach for predicting PPIs from protein primary sequences by a novel local conjoint triad feature representation with DNNs. The LCTD takes PPIs of continuous segments and discontinuous segments in protein sequence into account at the same time. The feature sets, characterized by LCTD, are capable of capturing more essential interactions information from the continuous and discontinuous binding patterns within a protein sequence. We then train a DNN with LCTD feature sets as inputs. Finally, the trained DNN is employed to predict the new PPIs. The experimental results indicate that DNN-LCTD is very promising for predicting PPIs and can be an available supplementary tool to other approaches.

The high prediction accuracy can be partially attributed to a biased selection of positive/negative training data. In practice, the available PPIs are incomplete and have a high rate of false positives and false negative. Furthermore, constructing the negative data set by subcellular localization information may also result in bias. How to construct a high quality negative set and how to reduce the impact of noisy and bias of PPIs data are future pursues. Another possible reason for the high accuracy is that DNN can model complex relationship between molecules by hidden layers and reduce the impact of noisy and bias of PPIs data.

## Figures and Tables

**Table 1:** Recommended parameters of DNN-LCTD in the experiments.

Name	Range	Recommendation
Learning rate	1,0.1,0.001,0.002,0.003,0.0001	0.002
Batch size	32,64,128,256,512,1024	512,1024
Weight initialization	uniform, normal, lecun_uniform, glorot_normal, glorot_uniform	glorot_normal
Per-parameter adaptive learning rate	SGD, RMSprop, Adagrad, Adadelta, Adam, Adamax, Nadam	Adam
Activation function	relu, tanh, sigmoid, softmax, softplus	relu, sigmoid
Dropout rate	0.5, 0.6, 0.7	0.6
Depth	2, 3, 4, 5, 6, 7, 8, 9	3
Width	16, 32, 64, 128, 256, 1024, 2048, 4096	2048, 512, 32
GPU	Yes, No	Yes

**Table 2:** Optimal parameters of comparing methods.

Method	Name	Parameters			
Guo’s work [19]	SVM + AC	C	γ		kernel
		32768.0	0.074325444687670064		poly
Yang’s work [20]	*k*NN + LD	n_neighbors	weights	algorithm	p
		3	distance	auto	1
Zhou’s work [33]	SVM + LD	C	γ		kernel
		3.1748021	0.07432544468767006		rbf
You’s work [21]	RF + MCD	n_estimators	max_features	criterion	bootstrap
		5000	auto	gini	True

SVM: support vector machine, *k*NN: *k*-nearest neighbor, RF: random forest, AC: auto covariance, LD: local descriptor, MCD: multi-scale continuous and discontinuous, rbf: radical basis function, gini: gini index.

**Table 3:** Results of five-fold cross validation on PPIs of *S. cerevisiae*.

Method		ACC	PE	RE	SPE	MCC	$F_{1}$	AUC
DNN-LCTD	fold 1	93.28%	93.35%	93.19%	93.37%	86.56%	93.27%	98.18%
	fold 2	93.22%	95.47%	90.78%	95.67%	86.55%	93.06%	97.99%
	fold 3	93.38%	93.74%	93.01%	93.75%	86.76%	93.37%	97.99%
	fold 4	93.10%	93.68%	92.60%	93.62%	86.21%	93.14%	97.74%
	fold 5	92.58%	92.52%	92.41%	92.75%	85.16%	92.47%	97.84%
	Average	93.11%±0.31%	93.75%±1.08%	92.40%±0.96%	93.83%±1.10%	86.24%±0.63%	93.06%±0.35%	97.95%±0.17%
Du’s work [22]	DNN + APAAC	92.58%±0.38%	94.21% ± 0.45%	90.95%±0.41%	94.41%±0.45%	85.41%±0.76%	92.55%±0.39%	97.55%±0.16%
You’s work [21]	RF + MCD	89.15%±0.33%	90.00%±0.57%	88.10%±0.17%	90.21%±0.61%	78.33%±0.67%	89.04%±0.31%	94.78%±0.21%
Zhou’s work [33]	SVM + LD	88.76%±0.37%	89.44%±0.27%	87.89%±0.45%	89.62%±0.30%	77.53%±0.53%	88.66%±0.28%	94.69%±0.31%
Yang’s work [20]	*k*NN + LD	84.81%±0.37%	87.53%±0.14%	81.18%±0.84%	88.44%±0.18%	69.80%±0.71%	84.23%±0.47%	90.03%±0.31%
Guo’s work [19]	SVM + AC	87.88%±0.56%	88.16%±0.90%	87.53%±0.59%	88.24%±1.02%	75.77%±1.12%	87.84%±0.53%	93.69%±0.33%

ACC: accuracy, PE: precision, SPE: specificity, MCC: matthews correlation coefficient, AUC: area under the receiver operating characteristic curve, DNN: deep neural network, RF: random forest, SVM: support vector machine, *k*NN: *k*-nearest neighbor, APAAC: amphiphilic pseudo amino acid composition, MCD: multi-scale continuous and discontinuous, LD: local descriptor, AC: auto covariance.

**Table 4:** Results on simulated *S. cerevisiae* dataset.

	ACC	PE	RE	SPE	MCC	$F_{1}$	AUC
fold 1	82.53%	92.24%	71.01%	94.04%	66.85%	80.24%	92.47%
fold 2	82.89%	93.57%	70.71%	95.12%	67.86%	80.55%	93.52%
fold 3	82.56%	93.25%	70.30%	94.89%	67.22%	80.16%	92.52%
fold 4	82.09%	94.02%	68.95%	95.52%	66.74%	79.56%	93.08%
fold 5	82.24%	91.74%	70.26%	93.86%	66.14%	79.58%	92.85%
Average	82.46%±0.31%	92.97%±0.95%	70.25%±0.79%	94.68%±0.71%	66.96%±0.64%	80.02%±0.44%	92.89%±0.43%

**Table 5:** Results based on DNNs with AC, LD, MCD, LCTD, CT, and APAAC on *S. cerevisiae* dataset. • indicates LCTD is statistically (according to pairwise *t*-test at 95% significance level) superior to the other descriptor.

	ACC (%)	PE (%)	RE (%)	SPE (%)	MCC (%)	$F_{1}$ (%)	AUC (%)
DNN-LCTD	93.11±0.33	93.75±0.88	92.40±0.81	93.83±0.85	86.24±0.66	93.06±0.39	97.95±0.16
DNN-MCD	91.35±0.31•	92.80±1.08	89.67±0.96•	93.03±1.10	82.76±0.64•	91.20±0.35•	95.10±0.17•
DNN-LD	90.19±0.26•	91.63±0.77•	88.46±0.42•	91.92±0.72•	80.43±0.55•	90.01±0.27•	96.33±0.18•
DNN-AC	89.49±0.36•	89.40±3.06•	89.61±3.92•	89.38±1.25•	78.99±1.19•	89.50±1.15•	95.89±0.31•
DNN-CT	91.84±0.31•	88.12±0.27•	79.81±1.08•	96.12±0.44	78.50±0.59•	83.65±0.46•	95.96±0.34•
DNN-APAAC	90.09±0.20•	91.66±0.27•	88.45±0.56•	91.77±0.33•	80.25±0.39•	90.03±0.23•	95.89±0.03•

**Table 6:** Comparison of training times of different comparing algorithms.

Method	DNN-LCTD (GPU)	DNN-LCTD (CPU)	SVM	*k*NN	Random Forest	Adaboost
Times (s)	718	2680	106,347	2814	6906	70,026

**Table 7:** Prediction results on five independent PPIs datasets, PPIs of *S. cerevisiae* are used as the training set.

Species	Test Pairs	ACC		
		DNN-LCTD	Du’s Work [22]	Zhou’s Work [33]
*C. elegans*	4013	93.17%	94.84%	75.73%
*E. coli*	6984	94.62%	92.19%	71.24%
*H. sapiens*	1412	94.18%	93.77%	76.27%
*H. pylori*	1420	87.38%	93.66%	75.87%
*M. musculus*	313	92.65%	91.37%	76.68%

**Table 8:** Division of amino acids into seven groups based on the dipoles and volumes of the side chains.

Group 0	Group 1	Group 2	Group 3	Group 4	Group 5	Group 6
A, G, V	C	F, I, L, P	M, S, T, Y	H, N, Q, W	K, R	D, E

## References

[1] Williams N.E. (2000). Immunoprecipitation procedures. Methods Cell Biol..

[2] Santoro C., Mermod N., Andrews P.C., Tjian R. (1988). A family of human CCAAT-box-binding proteins active in transcription and DNA replication: Cloning and expression of multiple cDNAs. Nature.

[3] Zhao X.M., Wang R.S., Chen L., Aihara K. (2008). Uncovering signal transduction networks from high-throughput data by integer linear programming. Nucleic Acids Res..

[4] Zhang Z., Zhang J., Fan C., Tang Y., Deng L. (2017). KATZLGO: Large-scale Prediction of LncRNA Functions by Using the KATZ Measure Based on Multiple Networks. IEEE/ACM Trans. Comput. Biol. Bioinform..

[5] Zhang J., Zhang Z., Chen Z., Deng L. (2017). Integrating Multiple Heterogeneous Networks for Novel LncRNA-disease Association Inference. IEEE/ACM Trans. Comput. Biol. Bioinform..

[6] Yu G., Fu G., Wang J., Zhao Y. (2017). NewGOA: Predicting new GO annotations of proteins by bi-random walks on a hybrid graph. IEEE/ACM Trans. Comput. Biol. Bioinform..

[7] Huang H., Alvarez S., Nusinow D.A. (2016). Data on the identification of protein interactors with the Evening Complex and PCH1 in Arabidopsis using tandem affinity purification and mass spectrometry (TAP–MS). Data Brief.

[8] Mehla J., Caufield J.H., Uetz P. (2015). Mapping protein-protein interactions using yeast two-hybrid assays. Cold Spring Harb. Protoc..

[9] Gavin A.C., Bösche M., Krause R., Grandi P., Marzioch M., Bauer A., Schultz J., Rick J.M., Michon A.M., Cruciat C.M. (2002). Functional organization of the yeast proteome by systematic analysis of protein complexes. Nature.

[10] Skrabanek L., Saini H.K., Bader G.D., Enright A.J. (2008). Computational prediction of protein-protein interactions. Mol. Biotechnol..

[11] Lee H., Deng M., Sun F., Chen T. (2006). An integrated approach to the prediction of domain-domain interactions. BMC Bioinform..

[12] Enright A.J., Iliopoulos I., Kyrpides N.C., Ouzounis C.A. (1999). Protein interaction maps for complete genomes based on gene fusion events. Nature.

[13] Aloy P., Russell R.B. (2002). Interrogating protein interaction networks through structural biology. Proc. Natl. Acad. Sci. USA.

[14] Aloy P., Russell R.B. (2003). InterPreTS: Protein Inter action Pre diction through T ertiary S tructure. Bioinformatics.

[15] Huang T.W., Tien A.C., Huang W.S., Lee Y.C.G., Peng C.L., Tseng H.H., Kao C.Y., Huang C.Y.F. (2004). POINT: A database for the prediction of protein-protein interactions based on the orthologous interactome. Bioinformatics.

[16] Du T. (2015). Predicting Protein-Protein Interactions, Interaction Sites and Residue-Residue Contact Matrices with Machine Learning Techniques.

[17] Bock J.R., Gough D.A. (2001). Predicting protein-protein interactions from primary structure. Bioinformatics.

[18] Shen J., Zhang J., Luo X., Zhu W., Yu K., Chen K., Li Y., Jiang H. (2007). Predicting protein-protein interactions based only on sequences information. Proc. Natl. Acad. Sci. USA.

[19] Guo Y., Yu L., Wen Z., Li M. (2008). Using support vector machine combined with auto covariance to predict protein-protein interactions from protein sequences. Nucleic Acids Res..

[20] Yang L., Xia J.F., Gui J. (2010). Prediction of protein-protein interactions from protein sequence using local descriptors. Protein Pept. Lett..

[21] You Z.H., Zhu L., Zheng C.H., Yu H.J., Deng S.P., Ji Z. (2014). Prediction of protein-protein interactions from amino acid sequences using a novel multi-scale continuous and discontinuous feature set. BMC Bioinform..

[22] Du X., Sun S., Hu C., Yao Y., Yan Y., Zhang Y. (2017). DeepPPI: Boosting Prediction of Protein-Protein Interactions with Deep Neural Networks. J. Chem. Inform. Model..

[23] Wang Y., You Z., Li X., Chen X., Jiang T., Zhang J. (2017). PCVMZM: Using the Probabilistic Classification Vector Machines Model Combined with a Zernike Moments Descriptor to Predict Protein-Protein Interactions from Protein Sequences. Int. J. Mol. Sci..

[24] Zeng J., Li D., Wu Y., Zou Q., Liu X. (2016). An empirical study of features fusion techniques for protein-protein interaction prediction. Curr. Bioinform..

[25] Peng H., Long F., Ding C. (2005). Feature selection based on mutual information criteria of max-dependency, max-relevance, and min-redundancy. IEEE Trans. Pattern Anal. Mach. Intell..

[26] Chou K.C. (2004). Using amphiphilic pseudo amino acid composition to predict enzyme subfamily classes. Bioinformatics.

[27] Angermueller C., Pärnamaa T., Parts L., Stegle O. (2016). Deep learning for computational biology. Mol. Syst. Biol..

[28] Asgari E., Mofrad M.R.K. (2015). Continuous Distributed Representation of Biological Sequences for Deep Proteomics and Genomics. PLoS ONE.

[29] Browne M.W. (2000). Cross-validation methods. J. Math. Psychol..

[30] Bewick V., Cheek L., Ball J. (2004). Statistics review 13: Receiver operating characteristic curves. Crit. Care.

[31] Akobeng A.K. (2007). Understanding diagnostic tests 3: Receiver operating characteristic curves. Acta Paediatr..

[32] Bengio Y. (2012). Practical recommendations for gradient-based training of deep architectures. Neural Networks: Tricks of The Trade.

[33] Zhou Y.Z., Gao Y., Zheng Y.Y. (2011). Prediction of protein-protein interactions using local description of amino acid sequence. Adv. Comput. Sci. Edu. Appl..

[34] Ben-Hur A., Noble W.S. (2006). Choosing negative examples for the prediction of protein-protein interactions. BMC Bioinform..

[35] Cortes C., Vapnik V. (1995). Support-vector networks. Mach. Learn..

[36] Cover T., Hart P. (1967). Nearest neighbor pattern classification. IEEE Trans. Inf. Theory.

[37] Breiman L. (2001). Random Forests. Mach. Learn..

[38] Collins M., Schapire R.E., Singer Y. (2002). Logistic Regression, AdaBoost and Bregman Distances. Mach. Learn..

[39] Xenarios I., Rice D.W., Salwinski L., Baron M.K., Marcotte E.M., Eisenberg D. (2000). DIP: The database of interacting proteins. Nucleic Acids Res..

[40] Shin C.J., Wong S., Davis M.J., Ragan M.A. (2009). Protein-protein interaction as a predictor of subcellular location. BMC Syst. Biol..

[41] Wei L., Ding Y., Su R., Tang J., Zou Q. (2017). Prediction of human protein subcellular localization using deep learning. J. Parallel Distrib. Comput..

[42] Davies M.N., Secker A., Freitas A.A., Clark E., Timmis J., Flower D.R. (2008). Optimizing amino acid groupings for GPCR classification. Bioinformatics.

[43] Tong J.C., Tammi M.T. (2007). Prediction of protein allergenicity using local description of amino acid sequence. Front. Biosci..

[44] Dubchak I., Muchnik I., Holbrook S.R., Kim S.H. (1995). Prediction of protein folding class using global description of amino acid sequence. Proc. Natl. Acad. Sci. USA.

[45] Min S., Lee B., Yoon S. (2016). Deep learning in bioinformatics. Brief. Bioinform..

[46] LeCun Y., Bengio Y., Hinton G. (2015). Deep learning. Nature.

[47] Nair V., Hinton G.E. Rectified linear units improve restricted boltzmann machines. Proceedings of the 27th International Conference on Machine Learning.

[48] Cotter A., Shamir O., Srebro N., Sridharan K. Better mini-batch algorithms via accelerated gradient methods. Proceedings of the Advances in Neural Information Processing Systems.

[49] Kingma D., Ba J. Adam: A method for stochastic optimization. Proceedings of the 3rd International Conference for Learning Representations.

[50] Hinton G., Vinyals O., Dean J. (2015). Distilling the knowledge in a neural network. Comput. Sci..

